# The International Nucleotide Sequence Database Collaboration

**DOI:** 10.1093/nar/gkv1323

**Published:** 2015-12-10

**Authors:** Guy Cochrane, Ilene Karsch-Mizrachi, Toshihisa Takagi, International Nucleotide Sequence Database Collaboration

**Affiliations:** 1 European Molecular Biology Laboratory, European Bioinformatics Institute (EMBL-EBI), Wellcome Genome Campus, Hinxton, Cambridge CB10 1SD, UK; 2 National Center for Biotechnology Information, National Library of Medicine, National Institutes of Health, Bethesda, MD 20894, USA; 3 DDBJ Center, National Institute for Genetics, Mishima, Japan

## Abstract

The International Nucleotide Sequence Database Collaboration (INSDC; http://www.insdc.org) comprises three global partners committed to capturing, preserving and providing comprehensive public-domain nucleotide sequence information. The INSDC establishes standards, formats and protocols for data and metadata to make it easier for individuals and organisations to submit their nucleotide data reliably to public archives. This work enables the continuous, global exchange of information about living things. Here we present an update of the INSDC in 2015, including data growth and diversification, new standards and requirements by publishers for authors to submit their data to the public archives. The INSDC serves as a model for data sharing in the life sciences.

## INTRODUCTION

The International Nucleotide Sequence Database Collaboration (INSDC; http://www.insdc.org) (1) is one of the largest, longest-standing partnerships championing public access to primary scientific data. It has spearheaded the establishment of standards, formats and protocols for the collection of nucleotide sequence data and metadata, and has provided the internationally recognised system of accession numbers for data submitters and scientific journals since the early 1980s. INSDC partners are (in alphabetical order): the DNA Data Bank of Japan (DDBJ; http://www.ddbj.nig.ac.jp/) at the National Institute for Genetics in Mishima, Japan; the European Nucleotide Archive (ENA, www.ebi.ac.uk/ena) at the EMBL European Bioinformatics Institute (EMBL-EBI) in Cambridge, UK; and GenBank (http://www.ncbi.nlm.nih.gov/genbank) at the National Center for Biotechnology Information (NCBI) in Bethesda, MD, USA.

The INSDC's policy (http://www.insdc.org/policy.html), first published in 2002, emphasises the collaboration's mandate to uphold free, unrestricted access to all of the data records their databases contain. Every day, INSDC partners capture, preserve, share and exchange a comprehensive collection of nucleotide sequence and associated information. Thanks to the falling costs of sequencing, INSDC handles a staggering volume of data (2,400 trillion bases in 2015) and develops new services to handle the changing landscape of data types generated using emerging high-throughput technologies. Its repositories are built to accommodate everything from raw data (i.e. next-generation sequencing reads (2) through assembly data, experimental design details, taxonomic information, functional annotation and information about the projects and biological samples associated with sequencing efforts.

All INSDC partners provide assembled sequences and annotations and are well synchronised in their wider activities, including routine data exchange, standard formats and sharing technology. The INSDC is responsive to the rapidly changing needs of the world's growing molecular-biology community, but its core mandate remains unchanged. This article presents a reaffirmation of INSDC policy and overview of recent activities of the global, public nucleotide sequence archives.

## INSDC POLICY

The core of the INSDC policy is maintaining public access to the global archives of nucleotide data generated in publicly funded experiments. A key instrument for this is submission as pre-requisite for publication in scholarly journals, a convention in which INSDC partners and publishers work together to ensure timely and smooth flow of data into repositories for release before, or at the time of, literature publication. The primary benefit of this is that scientists all over the world can access these records at any time to plan experiments, analyse published findings or support their critique. It also ensures that the author of the work receives the appropriate credit, and that this narrative context remains linked to underlying data that remain in perpetuity. All database records submitted to the INSDC remain permanently accessible as part of the scientific record.

INSDC partners do not themselves place restrictions on the redistribution or use of the data. Terms of use are available from each partner (DDBJ: http://www.ddbj.nig.ac.jp/intro-e.html#mission, ENA: http://www.ebi.ac.uk/about/terms-of-use, GenBank: http://www.ncbi.nlm.nih.gov/home/about/policies.shtml). The INSDC places responsibility for ensuring quality and accuracy firmly with the submitting authors, though the teams that maintain the databases provide a wealth of tools and support for submitters to achieve the best quality and organisation of content possible.

## HIGH STANDARDS

The INSDC could not operate without the standardisation of all deposited data. The consortium's work in this area focuses on harmonising syntactical representation, supporting minimum information efforts and providing annotation style recommendations for consistency and clarity. Guidelines, data structures and systematic vocabularies developed by the INSDC include the Feature Table Definitions document (http://www.insdc.org/documents/feature-table), the INSDC country list (http://www.insdc.org/country.html) and conventions in the description of experimental support for annotated features (http://www.insdc.org/recommendations-vocabulary-insdc-experiment-qualifiers).

The partners support standardisation efforts driven by the expert communities for which sequence data is an essential tool. This includes the ‘Minimum Information about any (x) Sequence’ standard (MIxS, (3), which is developed by the Genomic Standards Consortium (4), and the Minimum Contextual Data Checklist for pathogen surveillance data, which is developed by the Global Microbial Identifier (GMI) initiative. The MIxS relates to reporting on biological material provenance and experimentation procedure associated with genomes, metagenomes and marker gene sequences and has a particular importance in environmental genomics. The GMI checklist relates to instructions for genome-scale pathogen sequence submissions, enabling the global identification of microorganisms and, ultimately, detection of outbreaks and new pathogens (see http://bit.ly/mindatamatch).

Using a standardised, INSDC-agreed language, submitters now report missing values for mandatory descriptors with structure (http://www.ebi.ac.uk/ena/about/missing-values-reporting). Such enhanced information makes it easier to interpret these cases sensibly.

INSDC partners have developed submission systems that guide users through the deposition of sequences, annotations and contextual data. These systems incorporate validations to ensure that deposited data is of high quality.

Adherence to agreed data standards allows INSDC partners to develop complementary data-submission tools with the same essential reporting requirements, to exchange data on a daily basis and to present the same content in different ways according to local user needs.

## DOUBLING TIME

The INSDC assembled/annotated sequence dataset grew from 450 481 663 919 bases in September 2012 to 1 401 669 271 501 in September 2015 (see Table 1 and http://www.ebi.ac.uk/ena/about/statistics). This means that since 2012, this part of INSDC has trebled in size.

**Table 1.** Growth in INSDC assembled/annotated sequences, 2012–2015

Year	Nucleotides in assembled/annotated sequences
September 2012	450 481 663 919
September 2013	670 004 320 378
September 2014	997 958 152 853
September 2015	1 401 669 271 501

After several years of aggressive data growth, the doubling time of read data in the public archives is increasing. In other words, it is taking longer for the volume of raw data to double. The doubling time for raw data in October 2015 was 20.6 months. While this rate is slower than in previous years, it remains rapid and challenging in terms of managing network and data storage.

INSDC partners continue to exploit reference-based compression models (5). CRAM (http://www.ebi.ac.uk/ena/about/compression-policy) and cSRA (https://github.com/ncbi/sra-tools/blob/master/README.md) are supported in sequence databases at EMBL-EBI and NCBI, respectively. In addition, INSDC partners work with data provider communities to control the size of submitted datasets through improved data structuring and organisation.

**Figure 1.**
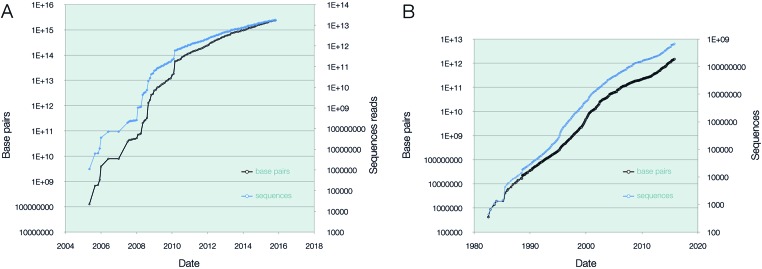
Cumulative growth in INSDC. (**A**) Base pairs (black, 2365.5 trillion) and sequence reads (blue, 17.8 trillion) for INSDC raw data. (**B**) Base pairs (black 1449 billion) and sequences (blue, 651.5 million) in INSDC assembled/annotated data.

In contrast, we have witnessed a sharp drop in doubling time for assembled/annotated sequence since 2013, representing an increase in growth rates for this section of INSDC. Figure 1 shows these rapidly increasing rates of growth in this area. We ascribe much of this effect to the embracing of whole-genome sequencing by the pathogen genomics community, covering surveillance, identification, typing and drug-resistance profiling and more exploratory research approaches for which data sharing and rapid access are vital.

Between January 2014 and October 2015, over 35 000 assembled prokaryotic and eukaryotic genomes were submitted to the INSDC databases.

## COLLABORATION

INSDC partners work in close collaboration with one another and with countless life-science communities throughout the world. The annual meetings of the INSDC address issues spanning day-to-day operations, specific details of the Feature Table Definitions document and questions of policy and strategy—notably in managing the rapidly growing volumes of sequence data that must be archived.
